# Empiric treatment of patients with sepsis and septic shock and place in therapy of cefiderocol: a systematic review and expert opinion statement

**DOI:** 10.1186/s44158-022-00062-7

**Published:** 2022-07-30

**Authors:** Andrea Cortegiani, Giulia Ingoglia, Mariachiara Ippolito, Massimo Girardis, Marco Falcone, Federico Pea, Francesco Pugliese, Stefania Stefani, Pierluigi Viale, Antonino Giarratano

**Affiliations:** 1 Department of Surgical, Oncological and Oral Science (Di.Chir.On.S.), University of Palermo, Palermo, Italy; 2 Department of Anesthesia, Intensive Care and Emergency, University Hospital Policlinico Paolo Giaccone, Palermo, University of Palermo, Via del Vespro129, 90127 Palermo, Italy; 3 Department of Anesthesia and Intensive Care, University Hospital of Modena, Modena, Italy; 4 Department of Clinical and Experimental Medicine, Infectious Diseases Unit, University of Pisa, Pisa, Italy; 5 Department of Medical and Surgical Sciences, Alma Mater Studiorum-University of Bologna, Bologna, Italy; 6 SSD Clinical Pharmacology Unit, University Hospital, IRCCS Azienda Ospedaliero Universitaria di Bologna, Bologna, Italy; 7 Department of Anaesthesiology Critical Care Medicine and Pain Therapy, Policlinico Umberto I, Sapienza University, Viale Regina Elena 324, 00161 Rome, Italy; 8 University of Catania, Department of Biomedical and Biotechnological Sciences, Biological Tower, Third floor -Est Tower, Catania, Italy; 9 Infectious Diseases Unit, Department of Medical and Surgical Sciences, Sant’Orsola Hospital, University of Bologna, Bologna, Italy

**Keywords:** Cefiderocol, Carbapenem resistance, Multidrug resistance, Gram-negative bacteria, Urinary tract infection, Nosocomial pneumonia

## Abstract

Carbapenem-resistant Gram-negative bacteria are frequent causes of sepsis and septic shock in intensive care unit (ICU) and thus considered a public health threat. Until now, the best available therapies consist of combinations of preexisting or new antibiotics with β-lactamase inhibitors (either new or preexisting). Several mechanisms of resistance, especially those mediated by metallo-β-lactamases (MBL), are responsible for the inefficacy of these treatments, leaving an unmet medical need. Intravenous cefiderocol has been recently approved by the American Food and Drug Administration (FDA) and European Medicines Agency (EMA) for the treatment of complicated urinary tract infections and nosocomial pneumonia due to Gram-negative, when limited therapeutical options are available. In addition, its ability to hijack bacterial iron uptake mechanisms makes cefiderocol stable against the whole Ambler β-lactamase inhibitors and increases the in vitro efficacy against Gram-negative pathogens (e.g., *Enterobacterales* spp., *Pseudomonas aeruginosa*, and *Acinetobacter baumannii*). Trials have already demonstrated their non-inferiority to comparators. In 2021, ESCMID guidelines released a conditional recommendation supporting the use of cefiderocol against metallo-β-lactamase-producing *Enterobacterales* and against *Acinetobacter baumannii*. This review provides the opinion of experts about the general management of empiric treatment of patients with sepsis and septic shock in the intensive care unit and detects the proper place in therapy of cefiderocol considering recent evidence sought through a systematic search.

## Introduction

Carbapenem-resistant Gram-negative bacteria are frequent causes of sepsis and septic shock in intensive care units (ICUs), where infections related to multidrug resistance (MDR) pathogens are dramatically increasing [1]. New strategies to improve outcomes and decrease mortality rates in hospitalized patients are urgently required. An early and effective treatment is essential to increase survival rate [2], but simultaneously the challenge of reducing the high rate of antibiotic resistance must be considered. Several mechanisms of resistance, indeed, especially those mediated by metallo-β-lactamases (MBL) are responsible for inefficacy of the best available treatments [3], leaving an unmet medical need. To improve outcomes of patients with MDR pathogen infections in ICU and reduce mortality, timely treatments are needed and empiric therapies, depending on intensive care epidemiology and patient’s medical history, must be administered early. An empiric therapy is based on a clinical hypothesis in the absence of a certain diagnosis. As a consequence of inappropriate empiric therapy, the spread of antimicrobial resistance both inside hospitals and into the community resulted to be extremely challenging to physicians [4]. The World Health Organization (WHO) has recently defined antimicrobial resistance as a public health threat. In addition, in 2017, WHO has already declared the priority on researching and investing on new therapies. Nowadays, the attention should be focused on some critical Gram-negative MDR pathogens: *Acinetobacter baumannii*, carbapenem-resistant *Pseudomonas aeruginosa*, carbapenem-resistant *Enterobacterales* (e.g., *Klebsiella pneumoniae*), and all the 3rd-generation cephalosporin-resistant bacteria [1, 5].

The significance of an antimicrobial stewardship program, characterized by a reasoned empiric therapy, based on the identification of the most common risk factors that can potentially lead to the identification of a pathological agent [6], and the value of a parsimonious usage of antimicrobials to prevent resistance were underlined. New antimicrobial combinations have been tested, both including non-β-lactamase inhibitors and β-lactamase inhibitors (e.g., ceftazidime-avibactam, ceftolozane-tazobactam, meropenem-vaborbactam, imipenem/cilastatin-relebactam) with partial success, because of the instability of beta lactamase ring or the non-susceptibility of some *A. baumannii* strains. Effective and timely treatments for MBL-producing Gram-negative-related infections are still an unmet medical need [7]; thus, new guidelines and new therapies have to be developed. In this scenario, cefiderocol turned out to be a valid option. Intravenous cefiderocol has been recently approved by the American Food and Drug Administration (FDA) and European Medicines Agency (EMA) for the treatment of complicated urinary tract infections and nosocomial pneumonia due to Gram-negative in case of limited therapy availability. Moreover, cefiderocol is stable against the whole Ambler β-lactamase inhibitors and has in vitro efficacy against Gram-negative pathogens (e.g., *Enterobacterales*, *P. aeruginosa*, and *A. baumannii*) thanks to the ability to hijack bacterial iron uptake mechanisms.

The aim of this review is to provide the opinion of experts about the general management of empiric treatment of patients with sepsis and septic shock in the intensive care unit and to identify the correct place in therapy of cefiderocol in light of recent evidence sought through a systematic search.

## Methods

**Fig. 1:**
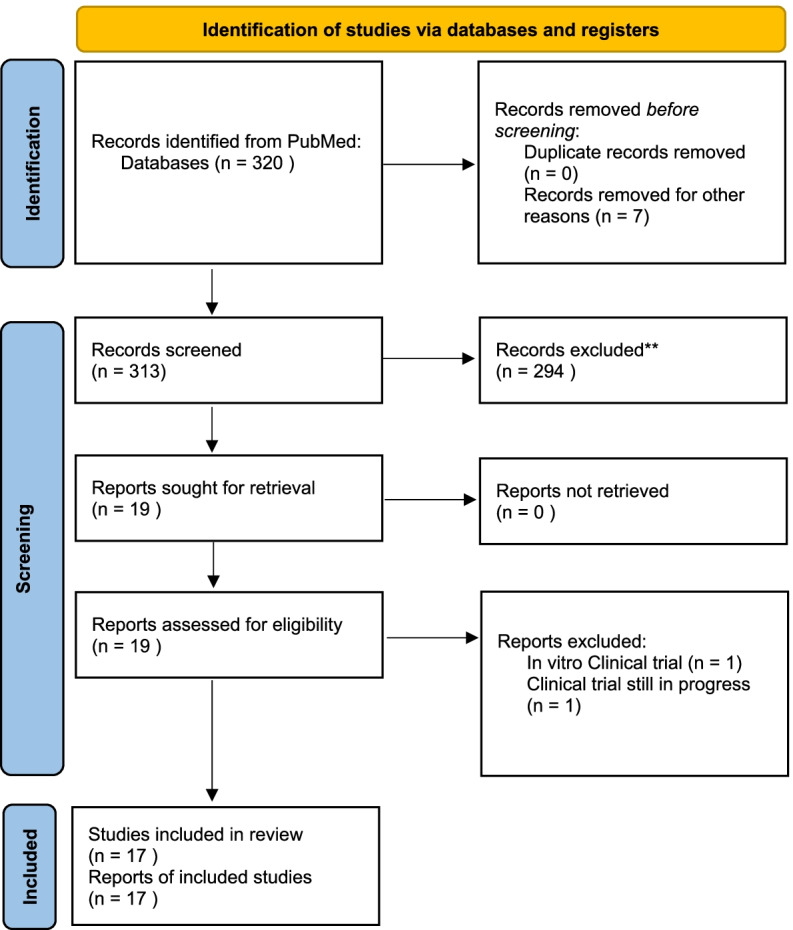
PRISMA 2020 flow diagram

A multidisciplinary team of experts was invited by one coordinator (AG) to join a focused discussion on key themes about (1) empiric treatment of patients with sepsis and septic shock and (2) place in therapy of cefiderocol, during two online meetings held by the Italian Society of Anesthesia Analgesia Intensive Care and Emergency (SIAARTI). During the same meetings, focused discussions among the panelists on the review topics were conducted. No standardized methods (e.g., analysis of agreement using voting results) for evaluating consensus among experts were adopted. For what concerns clinical data and the possible place in therapy of cefiderocol, a systematic review was conducted in accordance with the Preferred Reporting Items for Systematic Reviews and Meta-Analysis (PRISMA) statement [8]. A search was applied to the PubMed database using “Cefiderocol” as a keyword. Two authors (AC, GI) independently screened all titles and abstracts to select potentially eligible studies. Discrepancies at any stage were discussed and adjudicated in consensus. Randomized trials, both prospective and retrospective observational studies, meta-analysis, and guidelines were included. Reviews, case reports, and in vitro and non-clinical studies were excluded. In Fig. 1, the PRISMA flow diagram details the process of inclusion and exclusion. Only studies written in English were included.

## Results

From a total of 320 records screened, 19 were assessed for eligibility and 17 studies were included in the review.

### Cefiderocol: structure and mechanisms of action

Cefiderocol is a new siderophore cephalosporin, with demonstrated in vitro activity against Gram-negative microorganisms, including MDR pathogens, such as *A. baumannii*, *P. aeruginosa*, and *K. pneumoniae* [9]. Hijacking the bacterial iron-transfer mechanisms, cefiderocol can achieve high periplasmic concentrations. Its structure consists in a cephalosporin nucleus with two lateral chains similar to those of ceftazidime and cefepime. An aminothiazole ring and a carboxypropyl-oxymino group, situated in one of the two chains in C-7 position, simplify cefiderocol transport across Gram-negative bacterial outer membrane and enhance its stability against enzymatic β-lactamase hydrolysis.

The difference among cefiderocol, cefepime, and ceftazidime is given by a catechol moiety in C3 position; moreover, working as a siderophore, it creates a cefiderocol/ferric complex. Both passive diffusion and bacterial active-iron-uptake pumps allow cefiderocol to move across the outer bacterial membrane [9, 10]. Thanks to active transport, cefiderocol preserves the possibility to pass through cell membranes, even in case of permeability reduction or porin mutation and upregulation of efflux pumps [9, 10]. Once inside the periplasmic space, cefiderocol separates from iron and binds penicillin-binding protein (PBP), particularly in the form of PBP3, thus inhibiting peptidoglycan synthesis and leading to cell death. Besides, since Gram-positive and anaerobic bacteria growth is less dependent on active iron transport enzymes, cefiderocol turns out to be ineffective [7, 9].

### Pharmacokinetic and pharmacodynamic

Being a cephalosporin, cefiderocol activity is well described by time-dependent killing, since its efficacy is enhanced by prolonged and continued infusions (3-h infusion turned out to be more effective than 1-h infusion) [10]. Table 1 shows minimum inhibitory concentration (MIC) breakpoints stated by the Clinical and Laboratory Standards Institute (CLSI), American Food and Drug Administration (FDA), and European Committee on Antimicrobial Susceptibility Testing (EUCAST).

**Table 1:** MIC breakpoints stated by the Clinical and Laboratory Standards Institute (CLSI), American FDA, and European Committee on Antimicrobial Susceptibility Testing (EUCAST)

	CLSI			FDA			EUCAST		
Microorganism	S	I	R	S	I	R	S	I	R
*Enterobacterales*	< 4	8	> 16	< 4	8	> 16	< 2	--	> 2
*P. aeruginosa*	< 4	8	> 16	< 1	2	> 4	< 2	--	> 2
*A. baumannii*	< 4	8	> 16	< 1	2	> 4	NA	NA	NA
*S. maltophilia*	< 4	8	> 16	NA	NA	NA	NA	NA	NA

Breakpoint CLSI published in June 2018; update expected in 2021. Breakpoint FDA updated till September 2020. Breakpoint EUCAST published in April 2020

*I* intermediate, *NA* non-applicable, *R* resistant, *S* sensible, *MIC* minimum inhibitory concentration, *CLSI* Clinical and Laboratory Standards Institute, *FDA* Food and Drug Administration, *EUCAST* European Committee on Antimicrobial Susceptibility Testing

In vivo studies on murine models showed no evidence of insurgence of resistance mechanisms after repetitive doses of cefiderocol [11, 12].

In humans, cefiderocol showed a linear kinetic in a range of doses between 100 and 400mg. The excretion is mediated by the kidneys, while the liver is responsible for metabolism [7, 13]. Studies on human beings support the use of cefiderocol as a treatment for pulmonary, urinary tract, and bloodstream infections, sepsis included [4, 7].

### In vitro activity

Two trials, SIDERO 2014 [14] and SIDERO 2015 [15], compared cefiderocol in vitro activity to those of the most relevant available therapies.

SIDERO 2014 tested cefiderocol against several pathogenic agents such as *Enterobacteriaceae*, *P. aeruginosa*, *A. baumannii*, and *S. maltophila*. Isolates from North America center laboratories and European medical center laboratories were collected. The Clinical and Laboratory Standards Institute broth microdilution method was used. Moreover, iron-depleted conditions were necessary to mimic human tissues and fluid environments and to promote the induction of active ferric iron transporters. The in vitro activity was compared to those of colistin, meropenem, ceftazidime-avibactam, ceftolozane-tazobactam, cefepime, and ciprofloxacin. Cefiderocol turned out to be the best option having a MIC < 4μg/ml (eradication rates: *Enterobacteriaceae* spp. 99.9%; *P. aeruginosa* 99.9%; *A. baumannii* 97.6%; *S. maltophila* 100%). Results were consistent even for meropenem non-susceptible isolates (meropenem non-susceptible eradication rates: *Enterobacteriaceae* spp. 100%; *P. aeruginosa* 100%; *A. baumannii* 96.9%), thus superior to ceftolozane-tazobactam or ceftazidime-avibactam. In a low number of isolates, an elevated MIC for cefiderocol was observed. The responsible mechanisms are still unknown: downregulation of active iron transporters or mutation in the binding site for iron can be involved [14].

SIDERO 2015 continued collecting data and confirmed SIDERO 2014 results [15].

In conclusion, more than 99% of at least 18,000 Gram-negative *Bacillus* tested showed a minimum inhibitory concentration (MIC) for cefiderocol of < 4 μg/ml [14, 15], thus demonstrating an in vitro effectiveness of cefiderocol against a wide range of Gram-negative bacteria, including those producing the whole types of β-lactamases according to Ambler classification [7, 14–16].

Although cefiderocol showed a low in vitro activity against New Delhi metallo-β-lactamase (NDM)-producing bacteria, recent evidence demonstrated cefiderocol activity against *Enterobacterales*, *P. aeruginosa*, MBL-producing *A. baumannii*, and some NDM-producing *K. pneumoniae* strains [17]. In particular, a sublineage of ST147 *K. pneumoniae* producing NDM-1 was isolated. It was characterized by a complex resistome responsible for resistance to most antimicrobials except from cefiderocol and few others. The acquisition of a chimeric plasmid, indeed, which included genes for the siderophores yersiniabactin and aerobactin, granted cefiderocol activity [18]. A cefiderocol MIC value of < 4 μg/ml was observed, in addition to the superiority in the eradication of bacteria with carbapenem resistance (meropenem and imipenem MIC > 6 μg/ml) [15]. The ARGONAUT (Antibacterial Resistance Leadership Group Reference Group for testing of Novel Therapeutics) report analyzed the in vitro activity of cefiderocol and comparators, focusing on carbapenem-resistant isolates. Results showed a correlation between the activity of cefiderocol and β-lactam resistance mechanisms. While the activity of non-fermenting was independent of the presence of β-lactamases, the activity against *Enterbacterales* was affected by the presence of extended-spectrum β-lactamase (ESBL) or carbapenemases, even if no obvious association was identified [19].

Further studies are needed to explain resistance mechanisms, focusing particularly on those arising in non-fermenting organisms as well as on NDM enzymes and combinations of resistance mechanisms, such as iron transporter mutations [7].

### Cefiderocol: clinical data

Cefiderocol activity has been tested in two different non-inferiority trials in patients affected by complicated urinary tract infections (APEKS-cUTI) [20] or nosocomial pneumonia (APEKS-NP) [21] and in a phase III randomized, open, multicentric, descriptive study, whose aim was to compare cefiderocol to the best available therapy (BAT) in adults with complicated infection due to Gram-negative carbapenem-resistant bacteria (CREDIBLE-CR) [22].

APEKS-cUTI is a phase II, multicentric, double-blinded, parallel-group, non-inferiority trial, conducted in 67 hospitals among 15 countries including adult (> 18 years) patients diagnosed with complicated urinary tract infections (cUTI) with or without pyelonephritis or patients admitted to hospital with acute uncomplicated pyelonephritis.

All the included patients were randomized to receive 1-h intravenous infusion of cefiderocol 2 g or imipenem-cilastatin 1 g, three times a day, every 8 h for 7–14 days, adjusting doses according to kidney function, weight, or both. Cefiderocol turned out to have a comparable efficacy to imipenem-cilastatin. A total of 452 patients were enrolled (cefiderocol group *n* = 303; imipenem-cilastatin group *n* = 149), of whom 448 (*n* = 300 in the cefiderocol group; *n* = 148 in the imipenem-cilastatin group) received treatment. A total of 371 patients (*n* = 252 in the cefiderocol group; *n* = 119 in the imipenem-cilastatin group) were diagnosed with a Gram-negative pathogen. The clinical response and the microbiological response at the test of cure (TOC) at day 7 (± 2 days) after the end of treatment were valued as a primary endpoint that was achieved by a total of 248 patients (73% or *n* = 183/252 in the cefiderocol group; 55% or *n* = 65 in the imipenem-cilastatin group; with an adjustment treatment difference of 18.58%, 95% CI 8.23–28.92; *p* = 0.0004), proving the cefiderocol non-inferiority [20].

Cefiderocol was well tolerated, and mild secondary effects were registered (41% or *n* = 122/300 patients in the cefiderocol group; 51% or *n* = 76/148 patients in the imipenem-cilastatin group). The most frequent were gastrointestinal disorders, e.g., diarrhea, nausea and vomiting, and abdominal pain.

A further post hoc analysis showed superiority of cefiderocol for the treatment of urinary tract infections, since major bacteremia eradication rate and clinical cure/improvement rate than in the imipenem-cilastatin group (100% against 77.8%) were achieved [23].

Porthsmouth et al. conducted a further pilot investigation analysis to assess patients’ clinical outcomes after APEKS-cUTI trial. They interviewed 371 patients submitting a 14-element questionnaire related to eventual new symptoms that appeared during follow-up, graded in “absent,” “mild,” “moderate,” or “severe.” According to patients’ responses, clinical cure rates were 89.7% in the cefiderocol arm and 84.9% in the imipenem-cilastatin arm (adjustment treatment difference: 4.96%; 95% CI − 2.48–12.39). At the end of treatment (EOT), cefiderocol group patients’ symptoms were improved and the clinical failure rate assessed by investigators was very small [24].

Moreover, a systematic review compared six cUTI clinical trials analyzing six different antimicrobials: ceftolozane-tazobactam (ASPECT-cUTI), ceftazidime-avibactam (RECAPTURE), meropenem-vaborbactam (TANGO-1), plazomicin (EPIC), cefiderocol (APEKS-cUTI), and fosfomycin (ZEUS). All of the studies showed a rate of microbiological eradication of > 92%, thus demonstrating cefiderocol non-inferiority. Fosfomycin was the only exception, with a lower eradication rate (84%), due to high rates of resistance [25].

APEKS-NP is a randomized, double-blinded, parallel-group, phase III, non-inferiority trial, conducted in 76 centers among 17 countries in Asia, Europe, and the USA including patients affected by Gram-negative nosocomial pneumonia, ventilator-associated pneumonia, or healthcare-associated pneumonia (HCAP). Patients received a continuous 3-h infusion of cefiderocol 2 g or meropenem 2 g every 8 h for 7–14 day, extended till 21 days if required by clinical conditions. The dose of meropenem was mutually agreed by experts and FDA [21].

In addition, all patients received an intravenous infusion of linezolid 600 mg bid for 5 days. A total of 300 patients were enrolled; stratified according to age, renal function, APACHE II score, ventilation status, disease severity, baseline pathogen, and pathogen groups; and randomly assigned to the cefiderocol group (*n* = 148) or to the meropenem group (*n* = 152). Gram-negative pathogen infections were diagnosed in 86% of the whole number (*n* = 251). The most common microorganism isolated included *K. pneumoniae* (*n* = 92; 32%), *P. aeruginosa* (*n* = 48; 16%), *A. baumannii* (*n* = 47; 16%), and *E. coli* (*n* = 41; 14%). All-cause mortality at day 14 (primary endpoint) and at day 28 (secondary endpoint) was evaluated and cefiderocol non-inferiority was demonstrated (all-cause mortality at day 14: 12.4% for the cefiderocol group; 11.6% for the meropenem group). Similar results were observed at day 28. Cefiderocol turned out to be non-inferior to meropenem in critical nosocomial pneumonia since no relevant clinical differences were observed between the two groups, except for those patients affected by HCAP (more patients died in the cefiderocol group, 9 vs 2). In those cases where meropenem MIC was > 16 mcg/ml, cefiderocol showed efficacy (mortality at 14 days 0%; mortality at 28 days 20%). Finally, cefiderocol was well tolerated and as safe as other cephalosporin or carbapenem [21].

CREDIBLE-CR is a randomized, open, multicentric, parallel-group, phase III descriptive pathogen-focused trial, conducted in 95 centers among 16 countries in Africa, South America, Europe, and Asia including adult patients affected by nosocomial pneumonia, bloodstream infections, sepsis, complicated UTI, and evidences of carbapenem-resistant Gram-negative pathogen [22]. Patients received a 3-h intravenous infusion of cefiderocol 2 g every 8 h or the best available therapy (BAT). A different dose of cefiderocol 2 g every 6 h was administered to those patients with creatinine clearance > 120 ml/min. In case of pneumonia, bloodstream infections, UTI, or sepsis, cefiderocol could be combined with a second antibiotic (excluding polymyxin, cephalosporin, carbapenem). The estimated length of treatment was 7–14 days, although considering the possibility to extend the treatment to 21 days, if required by clinical conditions. The best available therapy was chosen in consideration of pathogens involved and site of infections. *A. baumannii*, *P. aeruginosa*, and *K. pneumoniae* were the three most common pathogens. The primary outcomes were clinical cure at test of cure (TOC) at day 7 (for patients affected by nosocomial pneumonia, bloodstream infections, or sepsis) and microbiological eradication (for those affected by UTI). A total number of 152 patients were enrolled (cefiderocol group *n* = 101; BAT group *n* = 51, of whom only 49 received treatment). The most common Gram-negative pathogens were *A. baumannii* (*n* = 54; 46%), *K. pneumoniae* (*n* = 39; 33%), and *P*. *aeruginosa* (*n* = 22; 19%). Cefiderocol resulted to have similar clinical and microbiological efficacy in all patients (nosocomial pneumonia: clinical cure achieved by 50% or *n* = 20/40 in the cefiderocol group; 53% or *n* = 10/19 in the BAT group; bloodstream infection or sepsis: clinical cure achieved by 43% or *n* = 10/23 in the cefiderocol group; 43% or *n* = 6/14 in the BAT group; UTI: microbiological eradication achieved by 53% or *n* = 9/17 in the cefiderocol group; 20% or *n* = 1/5 in the BAT group) [22]. Thus, the clinical and microbiological efficacy of cefiderocol were comparable to the best available therapy. Moreover, even higher microbiological eradication rates in case of complicated urinary tract infections were observed (53% vs. 20%). CREDIBLE-CR results also support cefiderocol as a valid option for the treatment of carbapenem-resistant infections, when limited therapies are available [22]. The safety profile was coherent with those of previous studies, except for increased mortality in those patients affected by *Acinetobacter-*related pneumonia. This evidence seems not to be related to the safety of cefiderocol and deserves further studies [22]. Increasing mortality seems to not include patients affected by UTI [7].

The presented rates resulted to be consistent with those of a systematic review which analyzed the 3 RCTs (APEKS-NP, APEKS-cUTI, and CREDIBLE-CR). No substantial differences between cefiderocol and the comparators were observed in the clinical efficacy (OR = 1.04, 95% CI 0.73–1.48), all-cause mortality at day 14 and day 28 (OR = 1.25, 95% CI 0.69–2.26; OR = 1.12, 95% CI 0.69–1.82), and microbiological response (OR = 1.44, 95% CI 0.84–2.47), with a higher microbiological eradication rate for *E. coli* (OR = 1.91, 95% CI 1.13–3.22) [26].

A post hoc analysis of the three trials (APEKS-cUTI, APEKS-NP, and CREDIBLE-CR) suggested that cefiderocol could be a valid option for Gram-negative infections, even if resistance to other antibiotics was observed. In particular, both bacteremia eradication rates and clinical cure/improvement rates in APEKS-cUTI resulted to be higher for cefiderocol than comparators (100% vs 77.8%) [23].

Consistent results were reported by a comparative study on COVID-19 patients, which compared cefiderocol to BAT in patients with bacteremia and nosocomial pneumonia due to *A. baumannii* [27]; 107 patients were enrolled: 42 treated with cefiderocol and 65 with other therapies (among others colistin). No differences in mortality at day 28 were reported (55% vs 58%, *p* = 0.706). On the contrary, a new observational monocentric Italian study has been published, comparing cefiderocol to other antimicrobial associations including colistin in patients affected by critical infections mediated by carbapenem-resistant *A. baumannii* (CRAB) [24]. The study included 124 patients (47 cefiderocol vs 77 colistin) and, after adjusting any possible bias with propensity score, it related cefiderocol to a minor mortality rate at day 30 (hazard ratio 0.44, *p* < 0.001). Patients with bacteremia resulted to benefit the most from cefiderocol therapy; besides, no difference in mortality was observed between patients affected by HAP/VAP [28].

Recently, data from a post hoc analysis about the two randomized trials CREDIBLE-CR and APEKS-NP were disseminated relating to the efficacy of cefiderocol in patients affected by MBL-producing Gram-negative mediated infections [17]. A clinical recovery of 70.8% of patients among cefiderocol group vs 40% in patients with other treatments was documented; concurrently, a microbiological eradication of 58.3% vs 30% and a mortality at day 28 of 12.5% vs 50% were observed [17]. Falcone et al. confirmed the previous results in their study conducted among 18 patients affected by infections due to MBL (NDM or VIM-beta lactamase)-producing *Enterobacterales* [29].

### How to use it: dose and administration

The recommended dose for patients with normal kidney function is 2g administered with a 3-h intravenous infusion every 8 h (Table 2). It is recommended to adjust the dose according to the patient’s kidney function [7, 9]. No recommendations for adjustment of dose in obese patients have been already stated, neither evidences of spread in cerebrospinal liquor have been demonstrated [9].

**Table 2:** Cefiderocol doses adjusted on kidney function

Kidney function	GFR	Dose rates
Augmented^a^	CG-CLCR, > 120 ml/min	2 g every 6 h, 3-h infusion
Normal	MDRD-eGFR, 90–120 ml/min/1.73 m^2^	2 g every 8 h, 3-h infusion
Mildly compromised	MDRD-eGFR, 60–90 ml/min/1.73 m^2^	2 g every 8 h, 3-h infusion
Moderately compromised	MDRD-eGFR, 30–60 ml/min/1.73 m^2^	1.5 g every 8 h, 3-h infusion
Critically compromised	MDRD-eGFR, 15–30 ml/min/1.73 m^2^	1 g every 8 h, 3-h infusion
End-stage renal disease	MDRD-eGFR, < 15 ml/min/1.73 m^2^	0.75 g every 12 h, 3-h infusion
Intermittent dialysis required		0.75 g every 12 h, 3-h infusion, (3rd) supplementary dose 0.75 g after hemodialysis (during hemodialysis days)
CRRT required	Dosed according effluent rate:	
	◦ ≤ 2 l/h	⇒ 1.5 g every 12 h
	◦ 2.1–3 l/h	⇒ 2 g every 12 h
	◦ 3.1–4 l/h	⇒ 1.5 g every 8 h
	◦ ≥4.1 l/h	⇒ 2 g every 8 h

*CG-CLCR* Cockcroft-Gault creatinine clearance, *eGFR* estimated glomerular filtration rate, *MDRD* Modification of diet in renal disease, *CRRT* continue renal replacement therapy

^a^Patients with hypermetabolic states due to sepsis

### Safety profile

Cefiderocol resulted safe and well tolerated in phase I studies on humans [30]. Adverse events registered among phase II and phase III studies were low or moderate entities and as frequent as adverse events related to the comparison arm.

Saisho et al. compared cefiderocol adverse events vs placebo in humans [30]. Cefiderocol single dose (100 mg vs 250 mg vs 500 mg vs 1000 mg vs 2000 mg vs placebo) and cefiderocol multiple dose (1000 mg 1st group vs 1000 mg 2nd group vs 2000 mg vs placebo) were tested.

The most common adverse events reported were:

- Nausea
- Diarrhea
- Cutaneous rush
- Headache
- Increase of liver enzyme (aspartate aminotransferase/alanine aminotransferase) level
- Hypokalemia

Anomalies related to iron homeostasis were not reported in any case of administration of cefiderocol [30].

This is demonstrated in a post hoc analysis conducted using data from the APEKS-NP study and analyzing efficacy and safety parameters, including those specific for iron homeostasis (e.g., iron, total iron binding capacity, hepcidin, transferrin saturation). The primary outcome was a rate of all-cause mortality (ACM) at day 14; ACM at day 28 was considered as a secondary outcome. Data about serum iron concentrations were available for 292 patients, of whom 242 (*n* = 117 in the cefiderocol arm; *n* = 125 in the meropenem arm) had low iron levels and 50 (*n* = 27 in the cefiderocol arm; *n* = 23 in the meropenem arm) had normal iron levels. Concerning patients with low iron levels, ACM at day 14 was 12.3% in the cefiderocol arm and 11.6% in the meropenem arm. These results were similar for those patients with normal iron serum level. Also, rates for ACM at day 28 were consistent with the previous results, thus demonstrating that baseline iron serum levels had no influence on cefiderocol efficacy and safety profile [31].

Moreover, Matsunaga et al. evaluated the rate of incidence of adverse events (AEs) reported in APEKS-cUTI, APEKS-NP, and CREDIBLE-CR. The incidence of AEs was lower in the cefiderocol group than in comparators, and no differences in iron homeostasis were observed. Particularly, adverse events appeared in 10.2% of patients receiving cefiderocol, < 1% reported critical accidents, and 1.5% interrupted treatment because of side effects developed [32]. According to a retrospective cohort study conducted by Bleibtreu et al., thrombocytopenia due to the treatment occurred in one patient, but disappeared after treatment discontinuation [33].

Furthermore, a randomized, double-blind, placebo-controlled, phase I study was conducted to assess the effects of cefiderocol on ECG parameters. No correlations with prolongation of the QTcF interval after administrations of cefiderocol were observed [34]. Cefiderocol is well tolerated also in patients with renal impairment. Katsube et al., in their open-label phase I study, compared subjects with various degrees of renal function with a healthy control group. A single intravenous dose of cefiderocol 1000 mg was administered. For those with hemodialysis (HD) dependence, cefiderocol was administered twice: once 1 h after HD conclusion and the second one approximately 2 h before the normal scheduled HD session. Vital signs and physical examinations were conducted to monitor eventual adverse events. No consistent differences in safety profile were observed between the two groups [35].

## Discussion

### Considerations from 2021 ESCMID guidelines for the treatment of infections caused by MDR Gram-negative bacilli

In 2021, the European Society of Clinical Microbiology and Infectious Disease (ESCMID) stated the new targeted therapy guidelines [36] (GLs; Table 3). These GLs were based on a systematic revision of the literature and on scientific evidences related to those infections caused by the 3rd-generation cephalosporin-resistant (3-GCR) *Enterobacteriaceae* spp., carbapenem-resistant *Enterobacterales* (CRE), and carbapenem-resistant *A. baumannii* (CRAB) (GLs approved by the European Society of Intensive Care Medicine (ESICM)) [36].

**Table 3:** The European Society of Clinical Microbiology and Infectious Disease (ESCMID) stated the new targeted therapy guidelines

Pathogen	Targeted therapy — critical illness	Targeted therapy — noncritical illness	Not recommended antimicrobial
3rd-generation cephalosporin-resistant *Enterobacteriaceae*	*Bacteremia and complicated infections:* **Carbapenems** (e.g., imipenem o meropenem) *Bacteremia in the absence of septic* *shock:* ertapenem **Conditional recommendation**, moderate quality of evidence	**Piperacillin-tazobactam, amoxicillin/clavulanic acid or quinolones** **Conditional recommendation**, moderate quality of evidence	**Tigecycline, cephamycin, cefepime** **Strong recommendation** or **conditional** r**ecommendation** against using, low quality of evidence
Carbapenem-resistant *Enterobacterales* (CRE)	**Meropenem-vaborbactam** or **ceftazidime-avibactam** **Conditional recommendation,** moderate and low quality of evidence (respectively)	Individualized (on patient and on infection site) old-generation antimicrobial **Aminoglycosides** if UTI **Conditional recommendation,** low quality of evidence	**Tigecycline** if BSI, HAP/VAP **Conditional recommendation against** using, low quality of evidence
Metallo-β-lactamase (MBL)-producing carbapenem-resistant *Enterobacterales* or resistant to any other antimicrobial	*Monotherapy*: **cefiderocol conditional recommendation**, weak quality of evidence *Combined therapy:* **ceftazidime-avibactam + aztreonam** **Conditional recommendation,** moderate quality of evidence	Old-generation antimicrobials, according to GCP	
Carbapenem-resistant *P. aeruginosa*	**Ceftolozane-tazobactam** if in vitro efficacy **Conditional recommendation**, very low quality of evidence	Individualized (on patient and on infection site) old-generation antimicrobial	
Carbapenem-resistant *A. baumannii*	**Ampicillin-sulbactam** If efficacy in vitro tested in HAP/VAP **Conditional recommendation**, very low quality of evidence *If non-susceptible to sulbactam:* **polymyxin** or high-dose **tigecycline** (if with in vitro activity) *If critical infection:* antimicrobial **combination** with in vitro tested activity (colistin, aminoglycoside, tigecycline, sulbactam) is suggested **Conditional recommendation**, very low quality of evidence		Combination **colistin + meropenem** and combination **colistin + rifampicin** **Strong recommendation** against using, moderate/high quality of evidence **Cefiderocol** **Conditional recommendation** against using, low quality of evidence

*BSI* bloodstream infection, *HAP* hospital-acquired pneumonia, *UTI* urinary tract infection, *VAP* ventilatory-acquired pneumonia

### Remarks on appropriate empiric therapy in ICU

**Fig. 2:**
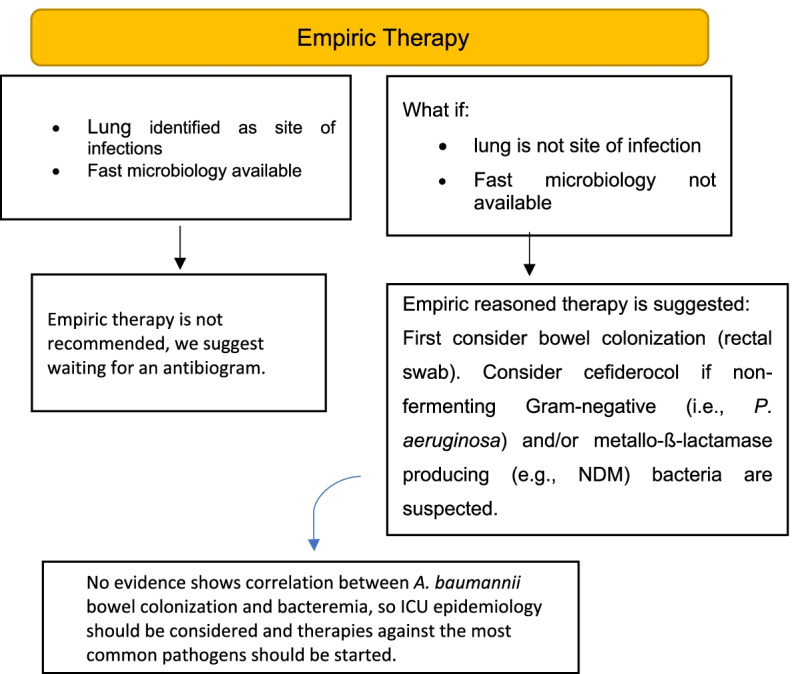
Suggestion on appropriate empiric treatment in patients with sepsis and septic shock

When sepsis occurs in ICU-hospitalized patients, timely strategies are needed to reduce mortality rate. As descripted before, if properly used, empiric therapy is an efficient resource, and an antimicrobial stewardship program should be used, in order to avoid resistance. We believe that, unless the patient is not affected by septic shock, physicians have enough time to obtain sufficient information about the suspected pathogens causing the infection. On the contrary, if not possible, physicians should consider infection site, colonization scenario, history of previous infections, or antimicrobial administrations and epidemiology to decide the best empiric therapy. According to the expert opinion, the patient’s medical history, physical examinations, and vital parameters can help physicians to suspect the more probable site of infection. When lungs are identified as the most probable, expert opinion suggests waiting for an antibiogram and to set up therapies according to it. On the contrary, when bacteremia occurs, and a rapid antibiogram is not available, or lungs are not considered the first site of the infection, a bowel colonization of *P. aeruginosa* should be suspected and an empiric therapy should be administered. Moreover, since *A. baumannii* bowel colonization is rarely responsible for developing a bacteremia, ICU epidemiology has to be evaluated and the more common pathogens have to be taken into account (Fig. 2).

However, the increasing spread of MDR pathogens requires more effective strategies and new perspectives of treatment. Due to its recent approval and the exiguity of published studies, the place in therapy of cefiderocol has to be still defined, thus conveying its strength in guidelines [7, 9]. Anyways, even more evidence is coming to light among literature, since recent clinical studies have demonstrated the important role that cefiderocol can play in the eradication of infections caused by Gram-negative MDR pathogens [17, 27–29, 37]. Beta lactamase antibiotics, as carbapenems, are still considered the best available therapy against Gram-negative extended-spectrum β-lactamase (ESBL)-producing pathogens. Besides, for those carbapenemase-producing bacteria, actual options are conditioned by several limits of efficacy or toxicity [7].

Cefiderocol is the first siderophore cephalosporin against Gram-negative bacteria approved in the USA and Europe Union (EU). Moreover, cefiderocol is the only one stable against all classes of β-lactamases, metallo-β-lactamase MBL included [7]. According to the Infectious Diseases Society of America (IDSA), cefiderocol is considered as one of the preferred options against NDM or other strains producing MBL and it is an alternative option in case of absence of certainty of carbapenemase resistance/presence of carbapenemase [38]. Cefiderocol has to be preferred in case of complicated UTI and pyelonephritis caused by carbapenem-resistant *Enterobacterales* (CRE) or by resistant and difficult-to-treat strains of *P. aeruginosa*. It has been also approved in case of hospital-acquired pneumonia and ventilator-associated pneumonia caused by *A. baumannii* complex, *E. coli*, *Enterobacter cloacae* complex, *K. pneumoniae*, *P. aeruginosa*, and *Serratia marcescens*. Moreover, real-world data support cefiderocol use in monotherapy or in combination with other antimicrobials as a rescue treatment for critical infections or immunocompromised patients.

Although the FDA approval of cefiderocol for cUTI was based on a randomized trial (APEKS-cUTI), data from CREDIBLE-CR concerning mortality were ambiguous. According to Naseer et al., several critical limitations affected this trial: a small sample size, the inclusion of cUTI with more serious infection, and a descriptive analysis without formal statistical testing. In addition, treatment failures progressing to sepsis and death were more frequent in the cefiderocol arm. For these reasons, the FDA convened to discuss the benefit/risk assessment of cefiderocol. The majority of the committee (14/16) confirmed the evidence of efficacy of cefiderocol for the treatment of cUTI. Besides, because of the mortality rate observed, a warning for use in indications other than cUTI is recommended [39].

Moreover, based on the data from CREDIBLE-CR and APEKS-NP trials, the ESCMID guidelines produced recommendations on the role of cefiderocol against infections by *A. baumannii.* The CREDIBLE-CR trial reported a numerically higher 28-day mortality rate in the cefiderocol group versus the BAT group among patients with infections by CRAB (49%, *n* = 19/39, vs 18%, *n* = 3/17). Indeed, in the trial, there was no advantage to cefiderocol with respect to clinical or microbiological eradication. Besides, in the APEKS-NP trial, similar mortality and clinical and microbiological eradication rates were observed between cefiderocol and high-dose extended-infusion meropenem in the small subgroup of patients (*n* = 47) with nosocomial pneumonia due to *A. baumannii*. Despite the limited information available, ESCMID issued a conditional recommendation (low certainty of evidence) against cefiderocol as treatment against infections caused by CRAB [21, 36].

Some observational studies also evaluated the efficacy of cefiderocol in critically ill patients affected by Gram-negative MDR infections, focusing on CRAB. Cefiderocol was used as a compassionate use in case of clinical failure (lack of clinical or microbiological improvement) with colistin on 10 intensive care patients affected by bacteriemia or ventilator-associated pneumonia caused by carbapenem-resistant Gram-negative bacteria, demonstrating a high percentage of success (70%) and a low rate of microbiological fail (20%). Only one case of induced resistance in vivo was observed [37]. Similar results were reported by Bavaro et al. The Italian team reported a case series of 13 patients (*n* = 5/13 critically ill patients with severe lung failure due to underlying Sars-Cov2 infection; *n* = 4/13 post-surgical infections; *n* = 4/13 severe infections in immunocompromised patients), reporting a rate of success for cefiderocol of 76.9%; the majority of the patients was affected by infections caused by *A. baumannii* [40]. In addition, a French retrospective cohort study reported the experience of cefiderocol as a salvage treatment after other antibiotics failure. Patients who received at least one dose of cefiderocol 2 g from November 2018 to November 2019 were included. The main sites of infection were the respiratory tract, followed by intra-abdominal, osteo-articular, skin-and-skin structures and the urinary tract. *P. aeruginosa*, *A. baumannii*, *K. pneumoniae*, and *Enterobacter hormaechei* were the pathogens isolated. A cure rate of 87.5% was observed in cefiderocol-susceptible isolate infections. Thrombocytopenia due to the treatment occurred in one patient, but disappeared after treatment discontinuation [33].

**Fig. 3:**
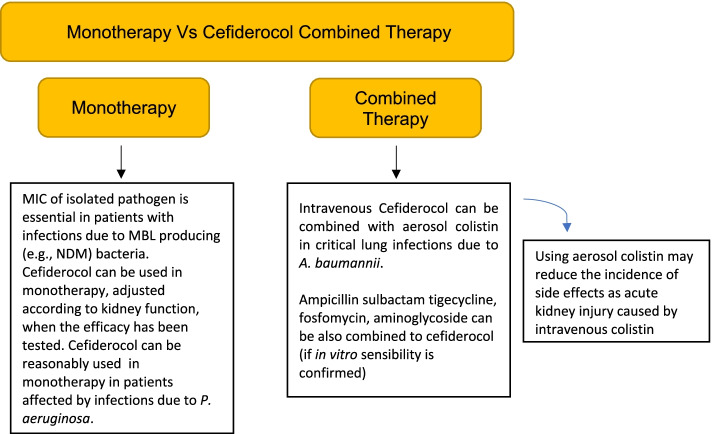
Suggestion: monotherapy vs combined therapy

Although not verified by clinical trial, there is the possibility to use cefiderocol in combination with aerosol colistin in case of *A. baumannii* lung infection in order to limit potential side effects on renal function of intravenous colistin. Further combinations of cefiderocol with antimicrobials such as tigecycline, ampicillin, and fosfomycin can be considered, too. Besides, when *P. aeruginosa* is isolated, cefiderocol can be reasonably administered in monotherapy and the kidney function must be considered. Moreover, when a MBL-producing pathogen infection is suspected, MIC has to be detected before administering cefiderocol (Fig. 3).

## Conclusions

Cefiderocol appears to be a new interesting therapeutical resource in case of failure of standard therapies and lack of alternatives, especially for what concerns nosocomial pneumonia and cUTI in mono or combined therapy. Indeed, it should be considered against proven or suspected infections due to non-fermenting Gram-negative, metallo-β-lactamase-producing bacteria and/or *A. baumannii*. In the latter case, the association with inhaled colistin or other antibiotics with confirmed susceptibility may be considered. Moreover, in case of septic shock, identifying the site of infection and an early identification of pathogens are essential to choose the best available therapy. Besides, when the first site of infection has not been identified, an empiric reasoned treatment is needed, focusing against CRAB and/or considering ICU epidemiology and patient’s risk factors.

## Data Availability

Not applicable.

## Abbreviations

3-GCR: 3rd-generation cephalosporin-resistant
ACM: All-cause mortality
AE: Adverse event
BAT: Best available therapy
Bid: Bis in die
BSI: Bloodstream infection
cUTI: Complicated urinary tract infections
CG-CLCR: Cockcroft-Gault creatinine clearance
eGFR: Estimated glomerular filtration rate
MDRD: Modification of diet in renal disease
CRRT: Continue renal replacement therapy
CLSI: Clinical and Laboratory Standards Institute
CRAB: Carbapenem-resistant *Acinetobacter baumannii*
CRE: Carbapenem-resistant *Enterobacterales*
ECG: Electrocardiogram
EOT: End of treatment
ESBL: Extended-spectrum β-lactamase
ESCMID: European Society of Clinical Microbiology and Infectious Disease
ESICM: European Society of Intensive Care Medicine
EU: Europe Union
EUCAST: European Committee on Antimicrobial Susceptibility Testing
FDA: Food and Drug Administration
GL: Guideline
HAP: Hospital-acquired pneumonia
HCAP: Healthcare-associated pneumonia
HD: Hemodialysis
ICU: Intensive care unit
IDSA: Infectious Diseases Society of America
MBL: Metallo-β-lactamase
MDR: Multidrug resistance
MIC: Minimum inhibitory concentration
NDM: New Delhi metallo-β-lactamase
NP: Nosocomial pneumonia
PBP: Penicillin-binding protein
PRISMA: Preferred Reporting Items for Systematic Reviews and Meta-Analysis
RCT: Randomized controlled trial
TOC: Test of cure
UTI: Urinary tract infection
VAP: Ventilatory-acquired pneumonia
VIM: Verona integron-encoded MBL inhibitor
WHO: World Health Organization
